# The Association between the Participation of Quality Control Circle and Patient Safety Culture

**DOI:** 10.3390/ijerph17238872

**Published:** 2020-11-29

**Authors:** Ni-Hu Tang, Shang-Feng Tsai, Jaw-Horng Liou, Yuan-Hui Lai, Shih-An Liu, Wayne Huey-Herng Sheu, Chieh Liang Wu

**Affiliations:** 1 Pharmacy Division, Taichung Veterans General Hospital Chiayi Branch, Chiayi 60090, Taiwan; tangwu@vghtc.gov.tw; 2 School of Medicine, National Yang-Ming University, Taipei 11221, Taiwan; s881056@gmail.com (S.-F.T.); an1654@seed.net.tw (S.-A.L.); 3 Division of Nephrology, Department of Internal Medicine, Taichung Veterans General Hospital, Taichung 40705, Taiwan; 4 Department of Life Science, Tunghai University, Taichung 40705, Taiwan; 5 Department of Pharmacy, Taichung Veterans General Hospital, Taichung 40705, Taiwan; liujh@vghtc.gov.tw; 6 School of Pharmacy, China Medical University, Taichung, Taiwan; 7 Center for Quality Management, Taichung Veterans General Hospital, Taichung 406040, Taiwan; yuanhuilai@vghtc.gov.tw; 8 Department of Otolaryngology, Taichung Veterans General Hospital, Taichung 40705, Taiwan; 9 Division of Endocrinology and Metabolism, Department of Medicine, Taichung Veterans General Hospital, Taichung 40705, Taiwan; whhsheu@vghtc.gov.tw; 10 Institute of Biomedical Sciences, National Chung Hsing University, Taichung 402, Taiwan; 11 School of Medicine, National Defense Medical Center, Taipei 114, Taiwan; 12 Department of Critical Care Medicine, Taichung Veterans General Hospital, Taichung 40705, Taiwan; 13 Department of Automatic Control Engineering, Feng Chia University, Taichung 40700, Taiwan; 14 Department of Industrial Engineering and Enterprise Information, Tunghai University, Taichung 40705, Taiwan

**Keywords:** quality control circle, patient safety culture, safety attitudes questionnaire, healthcare

## Abstract

Promoting patient safety culture (PSC) is a critical issue for healthcare providers. Quality control circles program (QCCP) can be used as an effective tool to foster long-lasting improvements on the quality of medical institution. The effect of QCCP on PSC is still unknown. This was a retrospective study conducted with matching data. A safety attitudes questionnaire (SAQ) was used for the evaluation of PSC. The association between all scores of six subscales of SAQ and the participation QCCP were analyzed with both the Mann–Whitney and Kruskal–Wallis tests. A total of 2718 valid questionnaires were collected. Most participants of QCCP were females (78.9%), nurses (52.6%), non-supervisors (92.2%), aged <40 years old (64.8%), degree of specialist or university graduates (78%), and with work experience of <10 years (61.6%). Of all participants, the highest scores were in the dimension of safety climate (74.11 ± 17.91) and the lowest scores in the dimension of working conditions (68.90 ± 18.84). The participation of QCCP was associated with higher scores in four dimensions, namely: teamwork climate (*p* = 0.006), safety climate (*p* = 0.037), perception of management (*p* = 0.009), and working conditions (*p* = 0.015). The participation or not of QCCP had similar results in the dimension of job satisfaction and stress recognition. QCCP was associated with SAQ in subjects with the following characteristics: female, nurse, non-supervisor, aged >50 years old, higher education degrees and with longer working experiences in the hospital. In this first study on the association between each dimension of SAQ and the implementation of QCCP, we found that QCCP interventions were associated with better PSC. QCCP had no benefits in the dimensions of job satisfaction and stress recognition.

## 1. Introduction

Promoting patient safety is a critical issue for healthcare providers. The World Health Organization has defined patient safety as “the prevention of errors and adverse effects to patients associated with healthcare” and “to do no harm to patients” [1]. Recently, patient safety is becoming a critical issue within healthcare organizations. The patient safety culture (PSC) of employees includes their shared beliefs, attitudes, values, norms, and behavioral characteristics. PSC influences attitudes and behaviors of staff in relation to patient safety performance [2]. It is well established that with a constant improvement in safety culture, medical errors could be reduced, leading to higher quality of healthcare [3,4]. Evidence showed that safety culture influences patient clinical outcomes, like rates of infection and readmission [5,6,7]. The safety attitudes questionnaire (SAQ) was developed by Sexton et al. [8] to evaluate PSCs widely. The three causal dimensions are teamwork climate, stress recognition, and perception of management. The three receiving dimensions are safety climate, job satisfaction, and working conditions [9]. The inter-relationships amongst these six dimensions were studied [9]. For example, teamwork climate and hospital management support for patient safety are two critical dimensions to improve PSCs, since these two dimensions have direct impacts on all six dimensions, with the exception of stress recognition [9]. However, it remains unclear as how to improve PSC in staff having different backgrounds.

Quality control circles program (QCCP) can be used as an effective tool to foster long-lasting improvement in the quality of medical institution. QCCP’s goal is to increase awareness of medical workers on spotting and solving medical problems, improving medical work environments, and eventually bettering safety, quality, and economic performance [10]. QCCP improves safety outcomes, such as facilitating incident reporting and reducing infections and other adverse events [5,11]. QCCPs have been the main focus of our institute, Taichung Veterans General Hospital (TCVGH), with the aim to improve service quality. From 2000 to 2019, our institute has conducted 30 QCCPs/year achieving good results [12,13,14]. Our QCCP activities included projects to reduce complications caused by venous indwelling needles, reducing internal errors from prescription dispensed for outpatients [10,15].

A variety of strategies have been used to improve PSC, like those in Taiwan from 2009 to 2016 [16]. Previous studies reported that three common types of interventions promote PSC: team training and team communication tools, executive walk rounds and interdisciplinary rounding, and comprehensive unit-based safety program [17]. The results showed that QCCP has prominent effects in long-lasting improvement in the quality of medical organizations [18]. However, the influence of QCCP on the PSC has been explored by only one study, in which the questionnaire of Hospital Survey on Patient Safety Culture (HSOPSC) was used as outcome after the establishment of QCCP [19]. In the present study, we aimed to determine if QCCP participation improves levels of PSC based on a larger case number and using another simpler evaluation tool on patient safety (safety attitude questionnaire (SAQ)).

## 2. Material and Methods

### 2.1. Characteristics of Taichung Veterans General Hospital (TCVGH)

This study was conducted in TCVGH, which is a 1500-bed medical center with around 5500 employees, located in Taichung City of Taiwan. As a public medical center, it aims to provide safe, high-quality medical services with advanced facilities and training programs as well as outstanding research and development programs. TCVGH is the referral hospital for the critically ill and difficult cases in central Taiwan. The case-mix index (levels of complexity/risk of disease and difficulty in treatment) of our patients is higher compared with other referral hospitals in Taiwan. In addition, it provides patient-centered holistic care with advanced equipment and technology. It involves multiple cross-department centers to provide integrated care services.

#### Center for Quality Management

Founded in July of 1986, TCVGH Quality of Medical Care Committee was chaired by the vice president of the institute and was responsible for the evaluation and management of issues concerning healthcare quality. Most departments and divisions formed their own Healthcare Quality Improvement Circles. They involved either a single unit or multidisciplinary team, to improve issues of healthcare quality including nursing care, environment safety, quality, and clinical process of patient care, and cross-department communications. The ultimate goal was to reduce medical costs, to improve patient satisfaction, and to better the public image of TCVGH.

### 2.2. Quality Control Circle program (QCCP)

**Figure 1:**
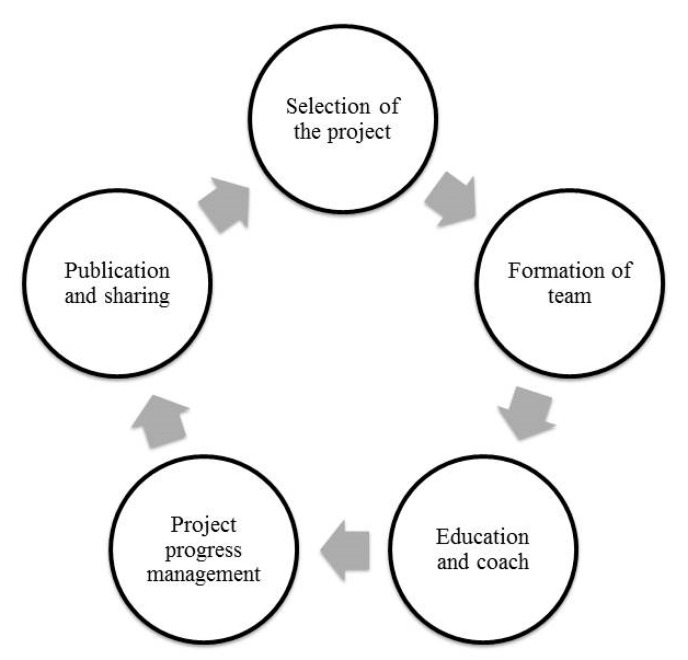
Annual activity plan of quality control circles program (QCCP).

**Figure 2:**
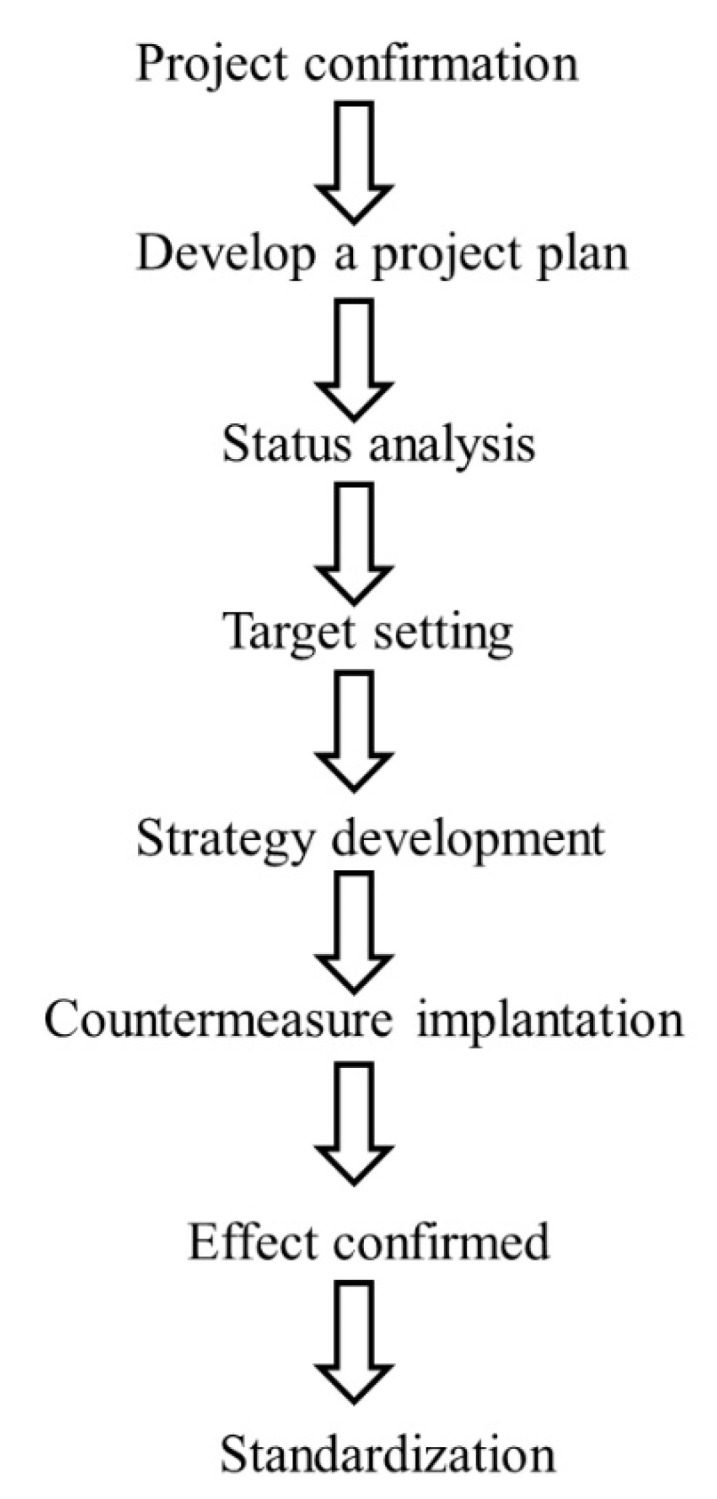
Flow chart of the project.

In 2000, our hospital started the comprehensive quality management, with the clinical department forming a QCCP to solve clinical problems to enhance the quality of healthcare. At the same time, the annual plan of QCCP was set up by the quality management center (Figure 1). This hospital arranged quality management-related education training for team members and provided expert guidance, and tracked the progress of each team based on the annual schedule of planning by the Quality Management Center (Figure 2). Each project was completed within a year, and results later published. T encouraged every project team translating results into the form of full papers to be published internationally at medical conferences.

### 2.3. Annula Survey of Safety Attitude Questionnaire (SAQ)

In 2009, our hospital implemented annual SAQ-based surveys regarding issues of patient safety. SAQ was first developed at the University of Texas. It is the one of the widely used instruments for PSC research in the healthcare industry [8,20,21]. SAQ contains 30 items to evaluate organizational staff’s opinions or attitudes toward PSC issues. It measures as already mentioned: namely teamwork climate, safety climate, job satisfaction, perception of management leadership, working conditions, and stress perception. Staff of the hospital (medical staff, and administrative personnel of all units, excluding short-term workers such as medical students and temporary workers) is typically asked to do the survey via an e-learning system. Each respondent rates 30 items according to a 5-point Likert scale ranging from strongly disagree to strongly agree, or a frequency such as “never, rarely, sometimes, most of the time and always”. The baseline data of subjects are collected for analysis, including job position (doctors, nurses, medical staff or administration staff), gender, supervisor or not, employment type (government employment or not), age, educational qualification, and years of experience in this hospital. Previous studies identified the above factors are associated with patient safety [21,22,23].

From 1 November 2018 to 30 November 2018, we conducted a cross-sectional survey using the SAQ questionnaire. At the time of completing the SAQ questionnaire, we also asked colleagues to indicate whether they had participated or not in the QCCP held in the hospital. Then, we analyzed possible effects from participating QCCP on their SAQ scores. All staff in this institute was encouraged to make response to this questionnaire. This survey had been performed from 2012 to 2020. This is voluntary only. The study protocol was approved by the Institutional Review Board of Taichung Veterans General Hospital in Taiwan (No: CW17045A). Informed consents of patients and family were waived, due to the pure data analysis nature of the study.

### 2.4. Statistical Methods

Frequency and descriptive statistics were generated for each variable in the questionnaire. Categorical data (i.e., profession, position, sex, educational level) were presented as frequency distribution and proportion. Numerical data (i.e., age, and years of work experience) were presented as mean ± standard deviation (SD). Initial data on variables of interest (i.e., job position, gender, supervisor, employment type, age, educational qualification and years of work experience in the hospital) between participating QCC or not were analyzed. The independent sample *t*-test was used to compare the means of continuous variables, and the *Chi*-square test was used to compare categorical variables between independent groups. After 1:1 data matching to reduce selection bias and to strengthen casual effects, we obtained a new database without any difference in the participation or not of QCCP across all variables of interest. After data matching, associations between scores of 6 subscales of SAQ and the participation of QCCP or not were determined using the Mann–Whitney and Kruskal–Wallis tests. The statistically significant difference was set at *p* < 0.05. The SPSS software (Statistical Package for the Social Science, version 20.0, Armonk, NY, USA) was used for all statistical analyses.

## 3. Results

### 3.1. Characteristics of Respondents

In this cross-sectional study, we collected 3939 questionnaires from staff. Initially, 2840 questionnaires were performed. After excluding invalid questionnaires (122 partially completed only), we finally analyzed a total of 2718 valid questionnaires. Of them, 502 employees had participated in the QCCP. All baseline demographic characteristics of participants are shown in Table 1. Most participants of QCCP were female (78.9%), nurses (52.6%), non-supervisors (92.2%), aged <40 years old (64.8%), holding degrees of specialist or university (78%), and with working experience <10 years (61.6%). All these baseline variables were associated with the participation of QCCP or not (*p* values all <0.05). However, after data matching, all baseline variables were not different between participation of QCCP or not.

### 3.2. Associations between QCC Implementation and Scores of SAQ

Table 2 shows mean difference of SAQ subscale between those with or without QCCP participation. Of them, total participants had the highest score in the dimension of safety climate (74.11 ± 17.91) and the lowest score in the dimension of working conditions (68.90 ± 18.84). After participation in QCCP, staff showed higher scores in the following four dimensions: teamwork climate (75.5 ± 18 vs. 72.4 ± 18.4, *p* = 0.006), safety climate (75.3 ± 17.9 vs. 73.0 ± 17.9, *p* = 0.037), perception of management (72.9 ± 19.5 vs. 70.1 ± 18.7, *p* = 0.009), and working conditions (70.4 ± 19.5 vs. 67.5 ± 18.1, *p* = 0.015). Participation of QCCP or not however, had no effect on the dimension of job satisfaction and stress recognition.

### 3.3. Associations between QCC Implementation and All Background Variables in the 6 Dimensions of SAQ

Associations between scores of the 6 dimensions of SAQ (with QCCP participation or not) and different baseline conditions are shown in Table 3. For the dimension of teamwork climate, staff showing higher scores were doctors, males, supervisors, public service workers, aged >50 years old, higher education degrees, and longer working experience in our hospital. In contrast, staff showing the lowest scores were administration staff, females, non-supervisors, non-public service workers, aged 31 to 40 years old, with lower education degree and shorter working experiences (5 to 10 years in our hospital). The participation of QCCP was associated with better teamwork climate in nurses (*p* = 0.016), females (*p* = 0.009), non-supervisors (*p* = 0.017), public service workers (*p* = 0.032), aged >40 years old (*p* = 0.042 for 41–50 years old and *p* = 0.036 for >50 years old), with higher education degrees (*p* = 0.001), and longer (>20 years) working experiences in our hospital (*p* = 0.012).

For the dimension of safety climate, staff having higher scores were doctors, males, supervisors, public service workers, aged >50 years old, with higher education degrees, and longer (>20 years) work experience. On the contrary, staff with the lowest scores was administration staff, females, non-supervisors, non-public service workers, aged 31 to 40 years old, with lower education degrees, and shorter work experience (5 to 10 years) in our hospital. The participation of QCCP was associated with better safety climate for administration staff (*p* = 0.018), non-supervisor (*p* = 0.032), aged 41 to 50 years old (*p* = 0.03), with higher education degrees (*p* = 0.009), and longer (>20 years) of work experience in our hospital (*p* = 0.018).

For the dimension of perception of management, staff with higher scores were doctors, males, supervisors, public service workers, aged >50 years old, with higher education degrees, and longer work experience (>20 years). On the contrary, staff with the lowest scores was administration staff, females, non-supervisors, non-public service workers, aged 31 to 40 years old, with lower education degrees, and shorter work experience (5 to 10 years) in our hospital. The participation of QCCP was associated with better perception of management in administration staff (*p* = 0.002), males (*p* = 0.049), non-supervisors (*p* = 0.019), public service workers (*p* = 0.031), aged >50 years old (*p* = 0.006), with higher education degrees (*p* = 0.002), and longer (>20 years) of work experience in our hospital (*p* = 0.014).

For the dimension of working conditions, staff with higher scores were doctors, males, supervisors, public service workers, aged >50 years old, with higher education degrees, and longer work experience (>20 years). On the contrary, staff with lowest scores was administration staff, females, non-supervisors, non-public service workers, aged 31 to 40 years old, with lower education degrees, and shorter work experience (5 to 10 years) in our hospital. The participation of QCCP was associated with better working conditions in males (*p* = 0.005), non-supervisors (*p* = 0.036), aged >50 years old (*p* = 0.004), with higher education degrees (*p* = 0.012), and longer work experience (>20 years) in our hospital (*p* = 0.001).

For the dimension of job satisfaction, staff with higher scores were doctors, males, supervisors, public service workers, aged >50 years old, with higher education degrees, and work experience of >20 years. On the contrary, staff with the lowest scores was administration staff females, non-supervisors, non-public service workers, aged <40 years old, with lower education degrees, and shorter work experience (1 to 10 years) in our hospital. The participation of QCCP did not affect job satisfaction under all conditions.

For the dimension of stress recognition, staff with higher scores were nurses, females, and aged 41 to 50 years old. On the contrary, staff with the lowest scores was administration staff (for QCC-) and doctors (QCC+), males, and those aged >50 years old. The participation of QCCP only improved stress recognition for those worked for 11 to 20 years in our hospital (*p* = 0.024).

## 4. Discussion

This study is the first one to investigate the association between SAQ and QCCP. The introduction of QCCP had not been integrated to improve patient care safety until 2018 [24]. Noviyanti et al. reported that implementation of quality control circle has a significant effect on patient safety and recommended implementing QCCP as a problem-solving approach to optimize patient safety [24]. In 2019, another study was also published to show a positive association between QCCP and HSOPSC [19]. Many safety culture surveys exist, but only two of them have been used extensively: HSOPSC [25] and SAQ [8]. SAQ and HSOPSC are similar in terms of reliability and predictive validity [26]. The implementation of QCCP produced higher HSOPSC scores (3.73 ± 0.61 vs. 3.57 ± 0.41, *p* < 0.05) based on a study with limited sample size [19]. The multiple linear regression of data in that study was only 0.407, indicating that the evidence is not compelling. There could be more independent variables. Thirdly, that study did not analyze the associations between each dimension of HSOPSC and QCCP. On the other hand, our present study had much more cases (2718 instead of their 685) for evaluating associations between each dimensions of PSC and the implementation of QCCP for quality improvement. Even their HSOPSC measures included more dimensions than the SAQ, that study failed to analyze each of the dimensions [19]. In addition, HSOPSC is more complicated than SAQ. SAQ is shorter, and one can evaluate PSC efficiently over a shorter time [26]. Our present study is the first one to elucidate the association between each dimension of SAQ and the implementation of QCCP.

Different characteristics of participants were associated with different results of SAQ. Of all scores of SAQ (Table 2), the result revealed that the highest score in the dimension of safety climate (74.11 ± 17.91) and the lowest score in the dimension of working conditions (68.90 ± 18.84). These results are associated with different background characteristics of participants in this study (Table 1): mostly females (78.9%), nurses (52.6%), non-supervisor (92.2%), younger than 40 years old (64.8%), with lower education degree (78%), and working period less than 10 years (61.6%). Those types of staff were younger and apt to obey orders. In the pre-employment training, we were enthusiastic on promoting PSC. The concept of safety climate was likely consolidated in their minds. In addition, in the past 10 years, we have been constantly promoting PSC. Therefore, most participants experienced the dimension of safety climate strongly. The lowest score was in the dimension of working conditions. That result is associated with more newly graduated nurses. For them, they had lower confidence in coping with patients, unfriendly senior staff, more sense of isolation and more pressure of burnout according to previous studies [27,28]. Kramer et al described the beginning work experience of new nurses as “reality shock” [28]. Therefore, they felt weaker support in working conditions, which was associated with lower scores in this dimension.

Different characteristics of participants were associated with different results of the implementation of QCCP. For all participants after the implementation of QCCP (Table 2), they made progress in four out of six dimensions of PSC (namely teamwork climate, safety climate, perception of management and working conditions). However, the participation of QCCP or not had no effect on the dimension of job satisfaction and stress recognition. There was still no effect on these two dimensions after the implementation of QCCP in all variables, including job position, gender, supervisor or not, employment type, age groups, education level, and working experience (Table 3). Some reasons can explain this result. The implementation of QCCP was requested by management unit and the topic should be associated with patient safety. Team members should include young staff in order to train them and adequately supervise them. All processes of QCCP were associated with four dimensions of SAQ (i.e., teamwork climate, safety climate, perception of management, and working conditions). The spirit of QCCP was not associated with job satisfaction and stress recognition. In addition, other more important factors (such as higher pay and salary, and psychological empowerment) are found to associate with job satisfaction [29]. QCCP alone therefore cannot improve this dimension significantly. As for stress recognition, it recognition influences job satisfaction but it is not associated with other dimensions, according to a study using causal relationship analysis [9].

For a variety of participants (i.e., doctor, male, supervisor, public service, older than 40 years of age, higher education degree, and longer than 20-year work experience in this hospital), they had significantly higher scores in nearly all dimensions (i.e., teamwork climate, safety climate, perception of management, working conditions, and job satisfaction) (Table 3). Even with such high scores, some characteristics (older than 50 years old, higher education degree and more experienced in this hospital) were still found to associate with improved scores after the implementation of QCCP

In the four former dimensions, the above characteristics (i.e., >50 years old, higher education degree, and more experienced in this hospital) indicated their greater dedication or faith to the institute. They were also more open-minded and more willing to join quality improvement. On the contrary, for those characteristics (non-doctor, female, and non-supervisor) associated with lower scores, they still showed improvements after the implementation of QCCP.

There are some limitations to this study. First, we did not record topics of QCCP. That could have affected the results of SAQ. Second, we did not analyze the association between all 29 items of SAQ and the implementation of QCCP. Thirdly, this is a retrospective study. We will perform another “before and after” study in the future, to investigate where a causal effect relationship between QCCP participation and improvement of SAQ. Finally, the performance of QCCP may also have affected the results. Despite of these limitations, the strength of our study is being the first one to demonstrate the association of all six dimensions of SAQ and the implementation of QCCP. The findings may help improving QCCP in terms of efficiency and feasibility.

## 5. Conclusions

Our study is the first one to elucidate the association between each dimension of SAQ and the implementation of QCCP. QCCP can be an intervention to improve PSC. To obtain greater benefits, one could focus on the special group of staff: female, nurses, non-supervisor, those older than 50 years of age, with higher education degrees, and more work experience in the hospital. However, the implementation of QCCP had no benefits on the dimension of job satisfaction and stress recognition.

## Figures and Tables

**Table 1:** Demographic characteristics of participants (before and after 1:1 matching).

	Before Matching							1:1 Matching						
	Total		QCC+		QCC−		*p* Value	Total		QCC+		QCC−		*p* Value
**Job Position**							0.037 *							0.823
Doctors	414	(14.90%)	79	(15.70%)	335	(14.70%)		169	(16.80%)	79	(15.70%)	90	(17.90%)	
Nurses	1462	(52.60%)	272	(54.20%)	1190	(52.20%)		534	(53.20%)	272	(54.20%)	262	(52.20%)	
Medical staff	473	(17.00%)	64	(12.70%)	409	(18.00%)		127	(12.60%)	64	(12.70%)	63	(12.50%)	
Administration staff	431	(15.50%)	87	(17.30%)	344	(15.10%)		174	(17.30%)	87	(17.30%)	87	(17.30%)	
**Gender**							0.001 **							1.000
Male	588	(21.20%)	134	(26.70%)	454	(19.90%)		268	(26.70%)	134	(26.70%)	134	(26.70%)	
Female	2192	(78.80%)	368	(73.30%)	1824	(80.10%)		736	(73.30%)	368	(73.30%)	368	(73.30%)	
**Supervisors**							<0.001 ***							1.000
Yes	218	(7.80%)	93	(18.50%)	125	(5.50%)		186	(18.50%)	93	(18.50%)	93	(18.50%)	
No	2562	(92.20%)	409	(81.50%)	2153	(94.50%)		818	(81.50%)	409	(81.50%)	409	(81.50%)	
**Employment Type**							<0.001 ***							0.948
Government employment	1458	(52.40%)	320	(63.70%)	1138	(50.00%)		642	(63.90%)	320	(63.70%)	322	(64.10%)	
Non-government employment	1322	(47.60%)	182	(36.30%)	1140	(50.00%)		362	(36.10%)	182	(36.30%)	180	(35.90%)	
**Age Group (Years)**							<0.001 ***							0.854
≤30 years Old	973	(35.00%)	131	(26.10%)	842	(37.00%)		266	(26.50%)	131	(26.10%)	135	(26.90%)	
31–40 years Old	829	(29.80%)	168	(33.50%)	661	(29.00%)		335	(33.40%)	168	(33.50%)	167	(33.30%)	
41–50 years Old	539	(19.40%)	119	(23.70%)	420	(18.40%)		228	(22.70%)	119	(23.70%)	109	(21.70%)	
>50 Years Old	439	(15.80%)	84	(16.70%)	355	(15.60%)		175	(17.40%)	84	(16.70%)	91	(18.10%)	
**Educational Qualification**							<0.001 ***							0.081
Specialist/University	2169	(78.00%)	322	(64.10%)	1847	(81.10%)		671	(66.80%)	322	(64.10%)	349	(69.50%)	
Bachelor’s degree and more	611	(22.00%)	180	(35.90%)	431	(18.90%)		333	(33.20%)	180	(35.90%)	153	(30.50%)	
**Years of experience in the hospital**							<0.001 ***							0.921
<1 year	289	(10.40%)	22	(4.40%)	267	(11.70%)		46	(4.60%)	22	(4.40%)	24	(4.80%)	
1 to 4 years	724	(26.00%)	110	(21.90%)	614	(27.00%)		219	(21.80%)	110	(21.90%)	109	(21.70%)	
5 to 10 years	701	(25.20%)	132	(26.30%)	569	(25.00%)		259	(25.80%)	132	(26.30%)	127	(25.30%)	
11 to 20 years	522	(18.80%)	133	(26.50%)	389	(17.10%)		259	(25.80%)	133	(26.50%)	126	(25.10%)	
>20 years	544	(19.60%)	105	(20.90%)	439	(19.30%)		221	(22.00%)	105	(20.90%)	116	(23.10%)	

*Chi*-Square test. * *p* < 0.05, ** *p* < 0.01, *** *p* < 0.001. QCC: quality control circle.

**Table 2:** Scores of safety attitudes questionnaire (SAQ) subscales between participants with or without QCC.

	Total (*n* = 1004)	QCC+ (*n* = 502)	QCC− (*n* = 502)	*p* Value
	Mean ± SD	Mean ± SD	Mean ± SD	
Teamwork climate	73.87 ± 18.53	75.54 ± 18.36	72.45 ± 18.30	0.006 **
Safety climate	74.11 ± 17.91	75.39 ± 17.76	72.91 ± 18.00	0.037 *
Perception of management	71.50 ± 19.16	72.88 ± 19.34	70.10 ± 18.75	0.009 **
Working conditions	68.90 ± 18.84	70.58 ± 19.51	67.50 ± 18.12	0.015 *
Job satisfaction	70.60 ± 21.07	71.13 ± 20.85	70.24 ± 20.90	0.484
Stress recognition	71.63 ± 21.68	71.01 ± 23.11	72.21 ± 20.21	0.736

Mann–Whitney test. * *p* < 0.05, **p < 0.01. QCC: quality control circle. SAQ: safety attitudes questionnaire.

**Table 3:** Scores of six subscales shown according to participants with or without QCC.

	Teamwork Climate			Safety Climate			Perception of Management			Working Conditions			Job Satisfaction			Stress Recognition		
	QCC+	QCC−	*p*	QCC+	QCC−	*p*	QCC+	QCC−	*p*	QCC+	QCC−	*p*	QCC+	QCC−	*p*	QCC+	QCC−	*p*
**Job Position**																		
Doctors	81.6	82.3		81.1	81.5		80.3	79.5		77.4	75.2		80.4	84.2		63.4	68.9	
	±17.8	±17.0		±17.3	±15.6		±18.2	±18.7		±18.7	±16.5		±19.2	±19.1		±28.1	±20.2	
Nurses	75.0	71.1	*	74.6	72.3		71.3	69.2		70.5	68.2		69.2	67.4		73.6	75.6	
	±18.4	±18.2		±17.5	±18.2		±19.0	±18.6		±19.2	±19.1		±20.6	±20.5		±20.7	±19.5	
Medical staff	73.1	72.1		74.7	73.2		70.5	68.3		64.4	62.8		69.3	68.1		72.5	72.1	
	±18.6	±15.9		±17.6	±16.0		±21.7	±17.0		±20.5	±14.9		±21.4	±18.7		±23.0	±20.6	
Administration staff	70.9	66.1		69.6	65.1	*	72.9	63.8	**	67.0	60.4		69.4	65.3		64.5	65.1	
	±15.8	±17.9		±18.6	±17.5		±16.0	±17.0		±18.3	±15.0		±20.5	±19.5		±25.5	±20.2	
*p*	++	++		+	++		++	++		++	++		++	++		++	++	
**Gende** **r**																		
Male	79.5	77.0		78.8	76.5		76.6	74.2	*	74.8	69.2	**	76.5	76.7		64.0	70.4	
	±17.9	±18.7		±17.8	±17.6		±20.5	±19.2		±19.6	±17.6		±20.9	±20.1		±27.5	±19.8	
Female	74.3	70.8	**	74.3	71.6		71.7	68.7		69.2	66.9		69.4	68.0		73.3	72.9	
	±18.4	±17.9		±17.6	±18.0		±18.8	±18.4		±19.3	±18.3		±20.6	±20.7		±21.0	±20.3	
*p*	**	**		**	**		**	**		**			**	**		**		
Supervisor																		
Yes	85.0	81.5		84.5	82.7		83.0	79.2		79.0	75.6		83.3	82.3		68.7	74.7	
	±15.1	±18.3		±16.3	±17.1		±17.7	±18.7		±18.5	±16.8		±17.2	±19.1		±24.6	±20.4	
No	73.4	70.5	*	73.3	70.9	*	70.6	68.2	*	68.7	65.8	*	68.3	67.7		71.5	71.7	
	±18.4	±17.7		±17.4	±17.5		±19.0	±18.2		±19.3	±17.9		±20.6	±20.4		±22.8	±20.2	
*p*	**	**		**	**		**	**		**	**		**	**				
**Employment Type**																		
Public service	77.1	73.7	*	77.1	74.5		75.4	72.4	*	72.3	70.0		73.3	73.1		71.8	72.7	
	±18.8	±18.9		±18.0	±18.5		±19.5	±19.5		±19.7	±19.1		±21.4	±20.8		±23.9	±21.1	
Non-official	72.8	70.3		72.3	70.1		68.2	66 ± 1		67.4	63.2		67.1	65.3		69.5	71.4	
	±17.3	±16.9		±17.0	±16.8		±18.2	6.6		±18.9	±15.3		±19.2	±20.1		±21.5	±18.6	
*p*	*	*		**	**		**	**		**	**		**	**				
**Age Group (Years)**																		
Under 30 Years Old	73.8	71.6		73.1	72.2		70.8	68.5		70.3	66.1		68.4	65.3		70.1	73.5	
	±16.7	±16.7		±16.4	±17.3		±18.1	±17.1		±18.6	±18.1		±19.5	±20.6		±20.9	±19.0	
3–40 Years Old	71.1	70.9		71.1	71.0		68.1	67.6		65.6	65.9		65.9	68.9		72.9	70.0	
	±19.1	±17.9		±18.1	±17.3		±18.5	±18.5		±19.4	±17.7		±20.8	±21.2		±22.5	±18.7	
41–50 Years Old	79.4	74.2	*	79.5	74.0	*	76.1	73.7		72.6	70.1		74.4	72.4		72.3	76.8	
	±17.7	±18.4		±15.8	±18.0		±18.6	±18.9		±18.4	±18.8		±19.6	±20.1		±24.2	±20.6	
Over 51 Years Old	82.0	74.4	*	82.3	76.4		81.6	72.8	**	78.2	69.5	**	82.0	78.0		67.0	68.6	
	±8.1	±21.2		±19.1	±20.0		±20.8	±20.6		±20.4	±17.7		±20.8	±19.6		±26.3	±23.2	
*p*	++			++			++	+		++			++	++			++	
**Educational Qualification**																		
Specialist/ University	72.7	71.7		72.3	71.9		69.1	69.0		68.1	66.6		67.6	68.7		71.5	71.3	
	±18.5	±17.9		±17.8	±17.4		±19.3	±17.9		±19.6	±17.5		±20.9	±20.8		±22.1	±20.4	
Bachelor’s degree and more	81.1	74.2	**	81.6	75.2	**	80.2	72.8	**	75.5	69.5	*	78.0	74.0		70.0	74.5	
	±16.8	±19.3		±16.1	±19.1		±17.3	±20.3		±18.4	±19.3		±19.1	±20.8		±25.0	±19.6	
*p*	**			**	*		**	*		**			**	**				
**Years of Experience in the Hospital**																		
Less than 1 year	75.1	69.2		79.1	70.4		70.6	69.8		69.7	64.1		72.8	67.0		71.9	67.7	
	±18	±19.4		±15.6	±20.1		±19.8	±18.0		±21.7	±17.6		±19.4	±21.5		±21.5	±19.6	
1 to 4 years	73.7	71.5		73.4	72.1		70.3	68.7		70.4	67.4		69.1	66.9		69.8	71.1	
	±16.0	±15.9		±16.1	±16.4		±18.4	±16.3		±19.1	±16.4		±19.4	±21.2		±21.7	±19.2	
5 to 10 years	72.2	72.3		71.3	72.1		70.1	68.6		66.4	64.7		66.4	68.2		70.8	74.8	
	±19.3	±18.5		±17.3	±17.6		±19.2	±19.1		±19.9	±18.5		±20.8	±21.1		±21.2	±16.9	
11 to 20 years	75.1	71.7		74.6	72.1		72.6	70.2		69.5	69.3		70.1	70.9		74.9	69.7	*
	±19.2	±18.8		±18.5	±18.6		±19.1	±19.9		±18.6	±19.1		±21.3	±21.5		±25.0	±20.9	
over 21 years	82.2	75.1	*	82.5	76.0	*	79.6	73.1	*	77.4	69.4	**	79.9	75.6		67.3	74.1	
	±17.2	±19.6		±17.5	±18.8		±19.5	±19.5		±18.8	±18.0		±19.8	±18.8		±24.2	±23.4	
*p*	+			+			++	+		++	+		+					

Mann–Whitney test. * *p* < 0.05, ** *p* < 0.01; Kruskal–Wallis test. + *p* < 0.05, ++ *p* < 0.01.
